# Investigation of multidrug-resistant ST2 *Acinetobacter baumannii* isolated from Saint George hospital in Lebanon

**DOI:** 10.1186/s12866-019-1401-2

**Published:** 2019-02-02

**Authors:** Tania Nawfal Dagher, Charbel Al-Bayssari, Selma Chabou, Nadine Antar, Seydina M. Diene, Eid Azar, Jean-Marc Rolain

**Affiliations:** 1 Aix Marseille Univ, IRD, APHM, MEPHI, IHU-Méditerranée Infection, Faculté de Médecine et de Pharmacie, 19-21 boulevard Jean Moulin, 13385 Marseille, Cedex 05 France; 2 Saint George Hospital University Medical Center, Faculty of Medicine and Medical Sciences, University of Balamand, Beirut, Lebanon; 3 Faculty of Sciences III, Lebanese University, Tripoli, Lebanon

**Keywords:** *Acinetobacter baumannii*, OXA-23, ST2 clone

## Abstract

**Background:**

*Acinetobacter baumannii* is an opportunistic pathogen causing various nosocomial infections. The spread of multidrug-resistant *A. baumannii* is a major public health problem. The aim of this study was to investigate the molecular epidemiology and the genetic support of multidrug-resistant *A. baumannii* isolates collected from Saint-Georges Hospital in Lebanon.

**Methods:**

Between January and August 2016, 31 *A. baumannii* isolates were collected from sputum samples of patients infected with ventilator-associated pneumonia (VAP) and treated with colistin-carbapenem combination therapy. Antibiotic susceptibility testing was performed using the disk diffusion method. Carbapenemases, extended spectrum β-lactamases encoding genes and *mcr-1/2* genes were investigated by RT-PCR and standard PCR. The epidemiological relatedness of the strains was studied using MLST analysis.

**Results:**

Most of the isolates exhibited multidrug-resistant phenotypes. All the isolates were carbapenem-resistant and among them, 30 carried the class D carbapenemase *bla*_oxa-23_ gene while one isolate carried *bla*_oxa-72_ gene. MLST results revealed three sequence types, namely ST2, ST699, and ST627. Isolates having ST2 were the most prevalent clone (29/31, 93.5%).

**Conclusions:**

This study shows a nosocomial spread of multidrug-resistant *A. baumannii* ST2 having *bla*_OXA-23_ gene in Saint-George in Lebanon. Monitoring and control measures need to be adopted to avoid the spread of *A. baumannii* to patients.

## Background

*Acinetobacter baumannii* is a glucose non-fermentative, gram-negative, opportunistic pathogen and is one of the leading causes of nosocomial and community infections [1, 2]. These features make *A. baumannii* capable of causing a wide variety of clinical complications such as pneumonia, particularly ventilator-associated pneumonia (VAP), bloodstream and urinary tract infections, meningitis, surgical site and wound infections especially in intensive care units [2, 3]. Carbapenems are the first choice in the treatment of severe *A. baumannii* infections [4]. Carbapenem resistant *A. baumannii* nosocomial outbreaks have become a major concern worldwide since they lead to limited treatment options [5]. Several mechanisms contribute to carbapenem resistance in *A. baumannii* such as the expression of β-lactamases, alteration of cell membrane permeability, increased expression of efflux pumps, DNA gyrases, and topoisomerases [1]. In addition, the presence of three different types of β-lactamases in *A. baumannii* leads to β-lactame resistance such as: (1) *bla*_GES-14,_ *bla*_TEM_*, bla*_SHV,_ *bla*_CTX-M_ and *bla*_KPC_ belonging to Ambler class A β-lactamases; (2) *bla*_IMP-like_, *bla*_VIM-like_, *bla*_SIM-1_, and *bla*_NDM-1_ belonging to metallo-β-lactamases and (3) *bla*_OXA-23-like_, *bla*_OXA-24-like_, *bla*_OXA-58-like,_ *bla*_OXA-143_, *bla*_OXA-235-like,_ and the intrinsic *bla*_OXA-51-like_, which are found by nature on the chromosome of *A. baumannii* and belonging to oxacillinases [6–8]. Oxacilinases usually do not hydrolyse carbapenems, although some of them produce resistance to carbapenem and are known as “Carbapenem hydrolyzing class D beta-lactamases” or CHDLs. The most common of these enzymes were identified in *Acinetobacter* spp., notably in *A. baumannii*, and are responsible for carbapenem resistance [9].

The global dissemination of *A. baumannii* have been described in several countries in the world such as those of Europe, America, Asia, the Middle East, Australia, and South Africa [10]. A study done by Flamm et al demonstrated that the Mediterranean regions and Europe have the highest frequency of MDR *A. baumannii* isolates [11]. For example, in South and Southeast Asia, the carbapenem-resistant *A. baumannii* is a major challenge in public health, where these strains are predominant in nosocomial infections [12]. Regarding Europe, a lower rate of carbapenem resistance was shown in France, Germany and Sweden (10–20, 8 and 4%, respectively) whereas the rates increase up to 50–80% in Turkey, 85% in Greece, 60% in Italy and 45% in Spain [13]. It has been noticed that in many cases, one or two epidemic strains were perceived in a certain epidemiological setting. This is due to the transfer of colonized patients who transmit these strains between hospitals [14]. To date, in Lebanon, there has been a higher level of carbapenem-resistant *A. baumannii* (CRAB) strains [15]. While the *bla*_OXA-24_ [16] and *bla*_OXA-58_ [17] have been identified in our country, the *bla*_OXA-23-like_ remains the most common among the CRAB isolates. Furthermore, the international clone ST2 was found to be broadly spread among this country [18]. At the same institution where this present study was done, the outbreak of *A. baumannii* began in 2010 as an epidemic with a high level of resistance due to the high usage of carbapanem [19]. Several studies showed the dissemination of CRAB isolates harboring mainly the *bla*_OXA-23-like_ and belonging to the international clone ST2 [20, 21] at Saint-George Hospital. The aim of the current study was to investigate the molecular epidemiology and the genetic support of multidrug-resistant (MDR) *A. baumannii* isolates collected from patients treated with colistin-carbapenem combination therapy in Saint-George Hospital, Beirut, to investigate if there is a change in the pattern of isolates recovered from patients at the same institution.

## Results

### Antimicrobial susceptibility testing of isolated strains

A total of 31 strains isolated from Saint-George Hospital in Beirut were identified by MALDI-TOF as *A. baumannii*. These isolates were collected from the sputum of the respiratory tract (Table 1). Antibiotic susceptibility testing results revealed high levels of resistance rates of all isolates to ticarcillin, ticarcillin clavulanic acid, piperacillin tazobactam, ceftazidime, cefepime, cefotaxime, imipenem, meropenem, ciprofloxacin, and levofloxacin. In addition, 3.2% of the isolates were resistant to gentamicin, tobramycin, and amikacin. E-tests showed high-level of resistance to imipenem, with MIC greater than 32 μg/ml for all the isolates. None of the isolates was resistant to colistin (MIC < 2 μg/ml).

**Table 1:** Phenotypic and genotypic features of 31 *Acinetobacter baumannii* isolated from Saint Georges hospital in Lebanon

Isolate	M / F	Collection date	Types of sputum	IPM	IMP MIC (μg/ml)	Carbapenemases	ESBL	MLST
1	M	10-06-16	Upper Respiratory Tract	R	> 32	OXA-23	TEM-1	ST2
2	M	06-07-16	Upper Respiratory Tract	R	> 32	OXA-23	TEM-1	ST2
3	M	08-06-16	Upper Respiratory Tract	R	> 32	OXA-23	TEM-1	ST2
4	F	23-07-16	Upper Respiratory Tract	R	> 32	OXA-23	TEM-1	ST2
5	M	10-06-16	Upper Respiratory Tract	R	> 32	OXA-23	TEM-1	ST2
6	M	08-08-16	Upper Respiratory Tract	R	> 32	OXA-23	TEM-1	ST2
7	M	20-06-16	Upper Respiratory Tract	R	> 32	OXA-23	TEM-1	ST2
8	F	01-07-16	Upper Respiratory Tract	R	> 32	OXA-23	TEM-1	ST2
9	M	15-06-16	Upper Respiratory Tract	R	> 32	OXA-23	TEM-1	ST2
10	F	07-07-16	Upper Respiratory Tract	R	> 32	OXA-23	TEM-1	ST2
11	F	06-02-16	Upper Respiratory Tract	R	> 32	OXA-23	TEM-1	ST2
12	F	30-03-16	Upper Respiratory Tract	R	> 32	OXA-23	TEM-1	ST2
13	M	16-03-16	Upper Respiratory Tract	R	> 32	OXA-23	TEM-1	ST2
14	F	15-02-16	Upper Respiratory Tract	R	> 32	OXA-23	TEM-1	ST2
15	M	11-03-16	Upper Respiratory Tract	R	> 32	OXA-23	TEM-1	ST2
16	M	02-05-16	Upper Respiratory Tract	R	> 32	OXA-23	TEM-1	ST2
17	M	14-01-16	Upper Respiratory Tract	R	> 32	OXA-23	TEM-1	ST2
18	M	28-01-16	Upper Respiratory Tract	R	> 32	OXA-23	TEM-1	ST2
19	M	06-02-16	Upper Respiratory Tract	R	> 32	OXA-23	TEM-1	ST2
20	M	08-02-16	Upper Respiratory Tract	R	> 32	OXA-23	TEM-1	ST2
21	M	24-03-16	Upper Respiratory Tract	R	> 32	OXA-23	TEM-1	ST2
22	F	28-01-16	Upper Respiratory Tract	R	> 32	OXA-23	TEM-1	ST2
23	M	31-01-16	Upper Respiratory Tract	R	> 32	OXA-23	TEM-1	ST2
24	M	08-03-16	Upper Respiratory Tract	R	> 32	OXA-23	TEM-1	ST2
25	M	08-03-16	Upper Respiratory Tract	R	> 32	OXA-23	TEM-1	ST2
26	F	27-01-16	Upper Respiratory Tract	R	> 32	OXA-23	TEM-1	ST699
27	M	31-01-16	Upper Respiratory Tract	R	> 32	OXA-23	TEM-1	ST2
28	F	30-01-16	Upper Respiratory Tract	R	> 32	OXA-23	TEM-1	ST2
29	M	16-02-16	Upper Respiratory Tract	R	> 32	OXA-23	TEM-1	ST2
30	M	12-01-16	Lower Respiratory Tract	R	> 32	OXA-23	TEM-1	ST2
31	M	12-01-16	Lower Respiratory Tract	R	> 32	OXA-72	TEM-1	ST627

*M* male, *F* female, *ESBL* extended spectrum ß-lactamases, *MLST* multilocus sequence typing, *ST* sequence type

### Detection of beta lactamase genes

Results of PCR for carbapenemase-encoding genes showed that 30/31 of the isolates harbored the acquired OXA carbapenemase *bla*_OXA-23-like_ and one isolate expressed the *bla*_OXA-24-like_ gene.

In addition the ß-lactamases gene *bla*_TEM_ was detected in all isolates. Using ARG-ANNOT, the analysis of the sequenced genes revealed that the *bla*_OXA-23-like_ encoded for the OXA-23 whereas the *bla*_OXA-24-like_ encoded for the OXA-72 variant. All the sequences of the *bla*_TEM_ gene were identified as *bla*_TEM-1_. None of the isolates harbored *bla*_NDM-1_, *bla*_OXA-58_, *bla*_VIM_ gene, *bla*_SHV_, *bla*_CTX-M_ and *mcr*-1, 2, 3, 4 and 5 genes*.*

### MLST analysis

MLST analysis showed that 93.5% (29/31) of the *A. baumannii* isolates belonged to ST2 sequence type, whereas two isolates were assigned to ST699 and ST627, respectively. The most common clone (ST2), harboring the *bla*_OXA-23_ and *bla*_TEM-1_ genes, was found to be circulating in the hospital. The isolate belonging to ST627 was associated with the production of the *bla*_OXA-72_ and *bla*_TEM-1_ genes (Table 1).

## Discussion

*Acinetobacter baumannii* has been identified as one of the most successful pathogens responsible for nosocomial infections especially for patients admitted to intensive care units (ICUs) [22]. *A*. *baumannii* is able to acquire resistance to broad types of antibiotics including carbapenems. Carbapenem-resistant *A. baumannii* has been reported worldwide and has become a significant health problem due to the limited options for antibiotic treatment [23, 24].

Between 1999 to 2009, carbapenem-resistant *A. baumannii* harboring the bla_OXA-58_ gene were predominant in the hospital flora of many Mediterranean countries such as Lebanon, Italy, Greece, and Turkey [25]. After 2009, a huge shift from OXA-58 *A. baumannii* to OXA-23 producing belonging to the international clonal I and II lineages has been observed globally [25].

An outbreak of MDR *A. baumannii* has been observed in Saint George Hospital in Beirut, Lebanon between November 2004 and October 2005 [17].

In our study, the *bla*_OXA-23_ gene was found in 30 carbapenem-resistant *A. baumannii* isolates (96.7%) recovered from Saint George hospital in Beirut. In 2015, a study done in Lebanon showed the predominance of Imipenem-resistant *A. baumannii bla*_OXA-23_ and *bla*_GES-11_ gene *-*among the majority of *A. baumannii*. This dissemination of OXA-23 carbepenemase in Lebanon is consistent with the worldwide epidemiology of OXA-23 [16]*.* Also in 2016, Al Atrouni et al. showed the high dissemination of carbapenem-resistant *A. baumannii* harboring the *bla*_OXA-23_ gene and belonging to the international clone II lineage [26] (Table 2).

**Table 2:** Study of carbapenemase *Acinetobacter baumannii* in Lebanon

Origine	Carbapenemase genes	Other resistant genes	ST	Ref
Clinical isolates	OXA-23, OXA-24, OXA-58, NDM-1		ST2, ST25, ST46, ST85, ST193, ST424, ST570, ST85, ST600, ST622, ST636, ST690, ST702, ST715, ST706, ST707, ST1, ST708, ST713, ST807, ST808, ST809, ST810, ST811 and ST812	[26]
Clinical isolates	OXA-23, OXA-24		ST2, ST4, ST10 and ST14	[20]
Clinical isolates	OXA-23, OXA-24, OXA-58			[30]
Clinical isolates	OXA-23, OXA-51, OXA66, OXA69, NDM-1		ST2, ST1, ST460, ST85, ST6, ST25, ST103, ST154, ST3, ST158, ST146, ST459, ST284, ST150, ST108, ST461, ST462	[31]
Clinical isolates	MBL, OXA			[46]
Clinical isolates	OXA-143		ST286 to ST296 and ST464 to ST476	[47]
Clinical isolates	OXA-71			[48]
Clinical isolates	OXA-23, OXA-24		ST2	[21]
Clinical isolates	OXA-58	GES-5		[49]
Clinical isolates	OXA-23, OXA-24	GES-11		[15]
Clinical isolates	OXA-23, OXA-24	GES-11		[50]
Livestock	OXA-23, OXA-58		ST491, ST492, ST493, ST2 and ST20	[51]

*ST* Sequence Type, *Ref* reference

All the isolates in our study were resistant to imipenem. In Lebanon in 2012, it has been shown that 88% of *A. baumannii* were imipenem-resistant. This number is closely related to the rates of 70% in Egypt, 24–72% in Turkey, 25–75% in Spain, and approximating 100% in Italy [16].

In addition to the *bla*_OXA-23_ gene, we identified the presence of the *bla*_OXA-24-like_ gene in one isolate. A study in Lebanon showed that there was a replacement of the predominant OXA-58 producing *A. baumannii* by OXA-23 producers and belonged to the International Clone II, using isolates recovered from patients from the same institution as those used in the present study, as well as there was the presence of *bla*_OXA-24-like_ gene in two isolates that harbored also the *bla*_OXA-23-like_ gene [20]. Our study revealed that the *bla*_OXA-24-like_ encoded for the OXA-72 variant, which was firstly reported in 2004 in an *A. baumannii* isolated from Thailand. After that the *Acinetobacter* spp. who carried this gene have been reported in different countries [27]. In Lebanon, the *bla*_OXA-72_ was firstly described in *A. calcoaceticus* isolated from purchased vegetables in Beirut [28]. It is also found in *A. pittii* isolated from patients admitted to Lebanese hospital in North of Lebanon, in 2015 [29]. Moreover, Rafei et al reported that among 31 carbapenem-resistant strains collected from different hospitals in Beirut and Northern Lebanon, 28 isolates carried the *bla*_OXA-23_ gene, 1 strain the *bla*_OXA-24_ gene and 2 strains the *bla*_OXA-58_ gene [30]. She also reported in 2015 the spread of the international clone II lineage with high incidence of *bla*_OXA-23_ carbapenemase, in addition to the presence of *bla*_NDM-1_, *bla*_OXA-51_, *bla*_OXA-66_ and *bla*_OXA-69_ in different hospitals in Tripoli, Lebanon [31].

Moreover, we found that the majority of our strains harbored the ß-lactamases *bla*_TEM-1_ gene. A study in Egypt in 2017 showed that *bla*_TEM_ is the most frequent gene for ESBL [32]. In Saudi Arabia, Aly et al revealed that some of the isolates harbored the *bla*_TEM_ resistance genes as well as the *bla*_PER-1_ gene [33] . Also in Turkey, a study by Beris showed that *bla*_TEM_ was the most prevalent ESBL type amongst *A. baumannii* strains isolated from different regions [34].

In our study, the analysis of MLST showed that the strains belonged to three different clones, ST2, ST699, and ST627, where the ST2 was the most common clone (29/31). The ST2 and ST699 clones were associated with the production of OXA-23 carbapenemase, and the clone ST627 was associated with OXA-72. The ß-lactamases (ESBL) *bla*_TEM-1_ gene was found in all STs clones (Table 1). It has been shown that the international complexes CC1, CC2, and CC3 account for the most *A. baumannii* infections around the world and are frequently related with the production of OXA-23-like, OXA-24-like, or OXA-58-like enzymes [35]. Moreover, a study was done by the SENTRY Antimicrobial Surveillance Program in six countries of the Asia-Pacific region such as China, Hong Kong, India, Korea, Singapore and Thailand showed that there was a high distribution of *A. baumannii* having the *bla*_OXA-23_ carbapenemase genes [36]. In addition, in the United States, the OXA-23 has been described as the major mechanism responsible to the resistance of *A. baumannii.* These outbreaks are mostly associated to the worldwide spread of the international clones I and II [37]. The ST2 clone has also been reported in several Mediterranean countries. From 1999 to 2009, a study in four Mediterranean countries (Greece, Italy, Lebanon and Turkey) showed that *A. baumannii* outbreaks were caused by the spread of strains belonging in particular to ST2 and, to a lesser extent to ST1, ST25, ST78 and ST20. These clones harbored the *bla*_OXA-58_, *bla*_OXA-23_ and *bla*_*O*XA-72_ genes [18]. In Greece, it has been observed that the ST2 was the most common clone circulating in Greek hospitals. These clones harbored the *bla*_OXA-23_ gene that was displacing the *bla*_OXA-58_ gene, which was the only carbapenemase found among carbapenem-resistant *A. baumannii* isolates until 2009 [38]. Moreover, the clone ST699, which belongs to the international clone CC2, was found for the first time in Lebanon but was reported in Beijing, China in a study that described the predominance of ST699 in 65 out of 97 clinical *A. baumannii* isolates collected from patients having nosocomial bloodstream infection [39]. In addition, the clone ST627, was also found for the first time in Lebanon, and was reported in Thailand in the database of Pasteur institute without a published paper.

## Conclusion

In conclusion, this study describes the detection of *bla*_OXA-23_, and *bla*_OXA-72_ genes in clinical isolates of *A. baumanii* in Lebanon. MLST showed that there was a major circulating clone ST2. In addition, the ST699 and ST627 clones had not been previously detected in Lebanon. The resistant *A. baumannii* isolates found in Saint George hospital in Lebanon in the present study belonged to international clone II lineage and harbored the *bla*_OXA-23_, which were firstly reported by Dahdouh et al and then again by Soudeiha et al in the same institution. Thus, it is clear that this clone has become endemic in the hospital and that an urgent strategy needs to be adopted to control the spread of such resistant microorganisms among patients as well as appropriate infection control measures and surveillance programs must be implemented. In addition, we believe that it is necessary to set up quality training for health professionals to promote a safe environment for staff, patients and visitors, in order that the risk of healthcare associated infections are kept to a minimum.

## Methods

### Bacterial isolates

Between January and August 2016, 31 *A. baumannii* were isolated from sputum of the respiratory tract of patients infected with ventilator-associated pneumonia (VAP) and receiving colistin-carbapenem combination therapy in Saint-George Hospital in Beirut. 29/31 samples were collected from the sputum of the upper respiratory tract and 2/31 from the sputum of the lower respiratory tract from hospitalized patients and kept at − 80 °C before being transported to the laboratory in Marseille. Once arrived, the isolates were cultivated for 24 h at 37 °C on Trypticase Sodium Agar medium (TSA). Colonies growing on this medium were identified using matrix-assisted laser desorption/ionization time-of-flight mass spectrometry (MALDI-TOF MS) (Microflex; Bruker Daltonics, Bremen, Germany) as previously described [40].

### Antibiotic susceptibility testing

The antibiotic susceptibility testing was performed using the disk diffusion method on Mueller-Hinton agar as recommended by the European Committee of Antimicrobial Susceptibility Testing (EUCAST) 2017. Fourteen different antibiotics were tested: ticarcillin, ticarcillin clavulanic acid, piperacillin tazobactam, ceftazidime, cefepime, imipenem, meropenem, gentamicin, amikacin, tobramycin, colistin, ciprofloxacin, levofloxacin, and cefotaxime. Interpretations of the results of antibiotic sensitivity testing were made according to EUCAST recommendations. In addition, E-test method (bioMérieux) was performed to determine the minimal inhibitory concentration (MIC) of imipenem as recommended by the 2017 European Committee of Antimicrobial Susceptibility Testing (EUCAST).

Moreover, the minimal inhibitory concentration (MIC) of colistin was determined using the broth microdilution method (Biocentric) according to EUCAST 2017.

### DNA extraction

Bacterial DNA was extracted using the automatic robot EZ1 (Qiagen BioRobot EZ1-, Tokyo, Japan), with the extraction kit (EZ1 DNA, Qiagen, Hilden, Germany), following the manufacturer’s instructions. The extracted DNA was eluted in 200 mL of elution buffer and was stored at − 20 °C.

### Screening of samples by real-time PCR and molecular characterization of beta lactamase genes

Real-time PCR was performed to screen for the presence of carbapenemase-encoding genes using specific primers previously described for *bla*_NDM-1_, *bla*_OXA-23_, bla_OXA-24_, *bla*_OXA-58_, *bla*_VIM_ and *bla*_SHV._ All MDR bacteria were also screened for β-lactamases (*bla*_CTX-M_, *bla*_TEM_, *bla*_SHV)_ genes and for the *mcr*-1, 2, 3, 4 and 5 genes as described previously [41–44]. Negative and positive controls were used in each assay. The positive PCR products for any gene tested were sequenced using BigDye1 terminator chemistry on an automated ABI 3130 sequencer (PE Applied Biosystems, Foster City, CA). The sequences of the genes obtained were analyzed using the ARG-ANNOT database [45] (http://backup.mediterranee-infection.com/article.php?laref=282&titre=arg-annot), and compared to other genes using the BlastN and BlastP of the National Center for Biotechnology Information (NCBI) database (https://blast.ncbi.nlm.nih.gov/BlastAlign.cgi).

### Multilocus sequence typing

Molecular typing of the isolates was done to determine the genetic relationship among the clinical isolates by using the seven housekeeping genes (*cpn60, fusA, gltA, pyrG, recA, rplB,* and *rpoB*) as described on Institute Pasteur’s MLST Web site (https://pubmlst.org/abaumannii). Each single locus has different allele and the allelic profile or sequence types (ST) of the seven loci were given a specific identification number.

## Abbreviations

AST: Antibiotic susceptibility testing
ESBL: Extended spectrum beta lactamase
MALDI-TOF: Matrix-assisted laser desorption and ionization time-of-flight mass spectrometry
MDR-AB: Multidrug-resistant *Acinetobacter baumannii*
MLST: Multilocus sequence typing
PCR: Polymerase chain reaction
RT-PCR: Real time polymerase chain reaction
ST: Sequence type
